# 1-Benzoyl-3-ethyl-3-phenyl­thio­urea

**DOI:** 10.1107/S1600536811004326

**Published:** 2011-02-12

**Authors:** Aisha A. Al-abbasi, Mohammad B. Kassim

**Affiliations:** a School of Chemical Sciences and Food Technology, Faculty of Science and Technology, Universiti Kebangsaan Malaysia, 43600 Bangi Selangor, Malaysia

## Abstract

In the title compound, C_16_H_16_N_2_OS, the conformation at the two partially double C—N bonds of the thio­urea unit is *E*. The amide group is twisted relative to the thio­urea fragment, forming a dihedral angle of 62.44 (16)°, and the two phenyl rings form a dihedral angle 75.93 (18)°. In the crystal, mol­ecules are linked by N—H⋯S hydrogen bonds, forming centrosymmetric dimers.

## Related literature

For related structures and background references, see: Al-abbasi *et al.* (2010 ▶); Hung *et al.* (2010 ▶).
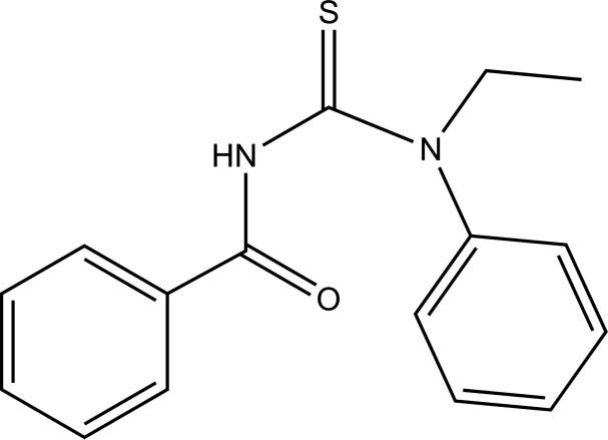


## Experimental

### 

#### Crystal data

C_16_H_16_N_2_OS
*M* *_r_* = 284.37
Triclinic, 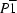

*a* = 7.735 (2) Å
*b* = 8.013 (2) Å
*c* = 12.540 (3) Å
α = 101.837 (5)°
β = 96.908 (5)°
γ = 94.205 (6)°
*V* = 751.3 (4) Å^3^
*Z* = 2
Mo *K*α radiation
μ = 0.21 mm^−1^
*T* = 298 K
0.53 × 0.38 × 0.19 mm

#### Data collection

Bruker SMART APEX CCD area-detector diffractometer
Absorption correction: multi-scan (*SADABS*; Bruker, 2000 ▶) *T*_min_ = 0.908, *T*_max_ = 0.961
7829 measured reflections
2648 independent reflections
2329 reflections with *I* > 2σ(*I*)
*R*_int_ = 0.023

#### Refinement

*R*[*F* ^2^ > 2σ(*F* ^2^)] = 0.054
*wR*(*F* ^2^) = 0.144
*S* = 1.06
2648 reflections
189 parameters
H atoms treated by a mixture of independent and constrained refinement
Δρ_max_ = 0.35 e Å^−3^
Δρ_min_ = −0.30 e Å^−3^

### 

Data collection: *SMART* (Bruker, 2000 ▶); cell refinement: *SAINT* (Bruker, 2000 ▶); data reduction: *SAINT*; program(s) used to solve structure: *SHELXS97* (Sheldrick, 2008 ▶); program(s) used to refine structure: *SHELXL97* (Sheldrick, 2008 ▶); molecular graphics: *SHELXTL* (Sheldrick, 2008 ▶); software used to prepare material for publication: *SHELXTL*, *PARST* (Nardelli, 1995 ▶) and *PLATON* (Spek, 2009 ▶).

## Supplementary Material

Crystal structure: contains datablocks I, global. DOI: 10.1107/S1600536811004326/gk2345sup1.cif

Structure factors: contains datablocks I. DOI: 10.1107/S1600536811004326/gk2345Isup2.hkl

Additional supplementary materials:  crystallographic information; 3D view; checkCIF report

## Figures and Tables

**Table 1:** Hydrogen-bond geometry (Å, °)

*D*—H⋯*A*	*D*—H	H⋯*A*	*D*⋯*A*	*D*—H⋯*A*
N1—H1*A*⋯S1^i^	0.83 (2)	2.62 (2)	3.444 (2)	172 (2)

Symmetry code: (i) 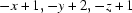.

**Fig. 1.**
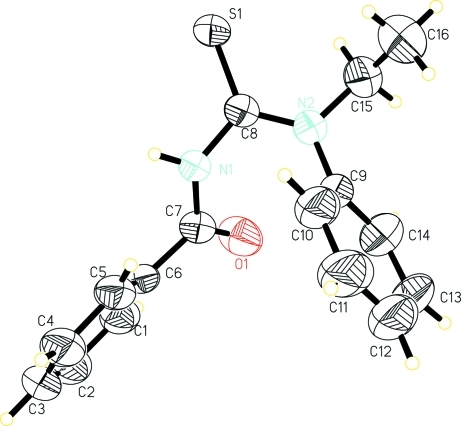
The molecular structure of the title molecule with displacement ellipsods drawn at the 50% probability level.

**Fig. 2.**
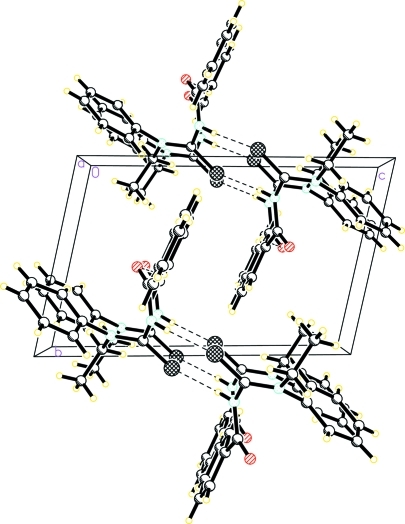
Crystal packing viewed down the a-axis. Hydrogen bonds are drawn as dashed lines.

## supplementary crystallographic information

### Comment

The ethyl group and S-atom are in *cis-*configuration with respect to the N2—C8 bond, however, the N2-phenyl and benzoyl groups are *trans* to S-atom with respect to both C—N thiourea bonds (Fig. 1).

The benzene ring [C1/C2/C3/C4/C5/C6/C7] (A), phenyl ring [N2/C9/C10/C11/C12/C13/C14] (B) and the thiourea [(S1/N1/N2/C8/] (C) fragment are essentially planar. The dihedral angle between the plane A and the plane B is 75.93 (15)° whereas, the dihedral angle between thiourea plane (C) and both planes (A, B) are 87.99 (11) and 62.44 (16)°, respectively. Furthermore, the amide group [N2/C9/C10/C11/C12/C13/C14] (D) is twisted relative to the thiourea fragment (C) forming a dihedral angle of 62.44 (16)°.

In addition, the molecules are linked by N1—H···S1 hydrogen bonds (Table 1, Fig. 2) to form centrosymmetric dimers.

### Experimental

The reaction scheme involved a reaction of benzoyl chloride (10 mmol) with ammonium thiocyanate (10 mmol) in acetone. The product, benzoyl isothiocyanate was reacted with *N*-ethylaniline (10 mmol) to give the title compound with 80% yield. A slow evaporation of the reaction mixture give light yellow crystals suitable for X-ray analysis.

### Refinement

Positions of C-bound H atoms were calculated. These H atoms were refined using a riding model with U_iso_=1.5U_eq_(C_methyl_ ) and U_iso_=1.5U_eq_(C) for the remaining H atoms. The amide-group H atom was located in a diffrence Fourier map and freely refined.

### Crystal data

**Table:**

C_16_H_16_N_2_OS	*Z* = 2
*M**_r_* = 284.37	*F*(000) = 300
Triclinic, *P*1	*D*_x_ = 1.257 Mg m^−^^3^
Hall symbol: -P 1	Mo *K*α radiation, λ = 0.71073 Å
*a* = 7.735 (2) Å	Cell parameters from 4273 reflections
*b* = 8.013 (2) Å	θ = 1.7–25.0°
*c* = 12.540 (3) Å	µ = 0.21 mm^−^^1^
α = 101.837 (5)°	*T* = 298 K
β = 96.908 (5)°	Blok, colorless
γ = 94.205 (6)°	0.53 × 0.38 × 0.19 mm
*V* = 751.3 (4) Å^3^	

### Data collection

**Table:**

Bruker SMART APEX CCD area-detector diffractometer	2648 independent reflections
Radiation source: fine-focus sealed tube	2329 reflections with *I* > 2σ(*I*)
graphite	*R*_int_ = 0.023
ω scan	θ_max_ = 25.0°, θ_min_ = 1.7°
Absorption correction: multi-scan (*SADABS*; Bruker, 2000)	*h* = −9→9
*T*_min_ = 0.908, *T*_max_ = 0.961	*k* = −9→9
7829 measured reflections	*l* = −14→14

### Refinement

**Table:**

Refinement on *F*^2^	Primary atom site location: structure-invariant direct methods
Least-squares matrix: full	Secondary atom site location: difference Fourier map
*R*[*F*^2^ > 2σ(*F*^2^)] = 0.054	Hydrogen site location: inferred from neighbouring sites
*wR*(*F*^2^) = 0.144	H atoms treated by a mixture of independent and constrained refinement
*S* = 1.06	*w* = 1/[σ^2^(*F*_o_^2^) + (0.0752*P*)^2^ + 0.3013*P*] where *P* = (*F*_o_^2^ + 2*F*_c_^2^)/3
2648 reflections	(Δ/σ)_max_ < 0.001
189 parameters	Δρ_max_ = 0.35 e Å^−^^3^
0 restraints	Δρ_min_ = −0.30 e Å^−^^3^

### Special details

**Table:**

Geometry. All e.s.d.'s (except the e.s.d. in the dihedral angle between two l.s. planes) are estimated using the full covariance matrix. The cell e.s.d.'s are taken into account individually in the estimation of e.s.d.'s in distances, angles and torsion angles; correlations between e.s.d.'s in cell parameters are only used when they are defined by crystal symmetry. An approximate (isotropic) treatment of cell e.s.d.'s is used for estimating e.s.d.'s involving l.s. planes.
Refinement. Refinement of *F*^2^ against ALL reflections. The weighted *R*-factor *wR* and goodness of fit *S* are based on *F*^2^, conventional *R*-factors *R* are based on *F*, with *F* set to zero for negative *F*^2^. The threshold expression of *F*^2^ > σ(*F*^2^) is used only for calculating *R*-factors(gt) *etc*. and is not relevant to the choice of reflections for refinement. *R*-factors based on *F*^2^ are statistically about twice as large as those based on *F*, and *R*- factors based on ALL data will be even larger.

### Fractional atomic coordinates and isotropic or equivalent isotropic displacement parameters (Å^2^)

**Table:**

	*x*	*y*	*z*	*U*_iso_*/*U*_eq_	
S1	0.23483 (8)	1.04861 (8)	0.44047 (5)	0.0590 (2)	
O1	0.2723 (2)	0.5506 (2)	0.26763 (17)	0.0716 (5)	
N1	0.4237 (2)	0.8089 (2)	0.34962 (15)	0.0467 (4)	
N2	0.2081 (3)	0.8877 (3)	0.23101 (16)	0.0663 (6)	
C1	0.5519 (4)	0.3888 (3)	0.3646 (2)	0.0629 (6)	
H1B	0.4418	0.3356	0.3670	0.076*	
C2	0.6996 (6)	0.3080 (4)	0.3850 (2)	0.0855 (10)	
H2A	0.6892	0.2010	0.4029	0.103*	
C3	0.8618 (5)	0.3839 (5)	0.3793 (2)	0.0900 (11)	
H3A	0.9606	0.3276	0.3922	0.108*	
C4	0.8789 (4)	0.5418 (5)	0.3545 (2)	0.0821 (9)	
H4A	0.9892	0.5925	0.3502	0.099*	
C5	0.7326 (3)	0.6268 (3)	0.33594 (19)	0.0567 (6)	
H5A	0.7447	0.7354	0.3204	0.068*	
C6	0.5686 (3)	0.5503 (3)	0.34046 (16)	0.0453 (5)	
C7	0.4073 (3)	0.6313 (3)	0.31503 (18)	0.0475 (5)	
C8	0.2875 (3)	0.9101 (3)	0.33425 (18)	0.0497 (5)	
C9	0.2909 (5)	0.8135 (4)	0.1373 (2)	0.0757 (9)	
C10	0.4569 (5)	0.8773 (4)	0.1275 (2)	0.0858 (9)	
H10A	0.5164	0.9661	0.1822	0.103*	
C11	0.5366 (7)	0.8103 (6)	0.0365 (3)	0.1170 (15)	
H11A	0.6502	0.8520	0.0317	0.140*	
C12	0.4498 (11)	0.6856 (8)	−0.0443 (4)	0.146 (2)	
H12A	0.5042	0.6398	−0.1044	0.175*	
C13	0.2835 (10)	0.6260 (7)	−0.0388 (3)	0.146 (2)	
H13A	0.2219	0.5451	−0.0975	0.175*	
C14	0.2019 (7)	0.6853 (6)	0.0554 (3)	0.1116 (15)	
H14A	0.092 (4)	0.660 (4)	0.063 (2)	0.067 (10)*	
C15	0.0405 (4)	0.9631 (4)	0.2081 (3)	0.0865 (9)	
H15A	−0.0407	0.8796	0.1550	0.104*	
H15B	−0.0115	0.9914	0.2754	0.104*	
C16	0.0708 (5)	1.1179 (5)	0.1650 (3)	0.1049 (11)	
H16A	−0.0382	1.1650	0.1512	0.157*	
H16B	0.1200	1.0893	0.0976	0.157*	
H16C	0.1506	1.2009	0.2178	0.157*	
H1A	0.499 (3)	0.850 (3)	0.403 (2)	0.050 (6)*	

### Atomic displacement parameters (Å^2^)

**Table:**

	*U*^11^	*U*^22^	*U*^33^	*U*^12^	*U*^13^	*U*^23^
S1	0.0635 (4)	0.0613 (4)	0.0516 (4)	0.0281 (3)	0.0083 (3)	0.0032 (3)
O1	0.0501 (9)	0.0559 (10)	0.1014 (14)	0.0015 (8)	0.0001 (9)	0.0070 (9)
N1	0.0474 (10)	0.0455 (10)	0.0438 (10)	0.0136 (8)	−0.0009 (8)	0.0026 (8)
N2	0.0700 (13)	0.0692 (13)	0.0521 (11)	0.0319 (10)	−0.0102 (9)	−0.0027 (9)
C1	0.0841 (17)	0.0513 (13)	0.0569 (14)	0.0164 (12)	0.0139 (12)	0.0139 (11)
C2	0.134 (3)	0.0642 (17)	0.0625 (17)	0.0491 (19)	0.0039 (17)	0.0151 (13)
C3	0.099 (2)	0.097 (2)	0.0681 (18)	0.062 (2)	−0.0074 (16)	−0.0040 (16)
C4	0.0544 (15)	0.103 (2)	0.0796 (19)	0.0283 (15)	0.0084 (13)	−0.0085 (17)
C5	0.0528 (13)	0.0592 (13)	0.0578 (13)	0.0148 (10)	0.0140 (10)	0.0049 (11)
C6	0.0545 (12)	0.0439 (11)	0.0376 (10)	0.0146 (9)	0.0101 (8)	0.0036 (8)
C7	0.0476 (12)	0.0452 (11)	0.0505 (12)	0.0088 (9)	0.0110 (9)	0.0086 (9)
C8	0.0499 (11)	0.0465 (11)	0.0509 (12)	0.0119 (9)	0.0031 (9)	0.0061 (9)
C9	0.109 (2)	0.0675 (16)	0.0456 (13)	0.0460 (16)	−0.0089 (14)	0.0000 (12)
C10	0.130 (3)	0.0819 (19)	0.0523 (15)	0.043 (2)	0.0186 (16)	0.0165 (14)
C11	0.183 (4)	0.120 (3)	0.070 (2)	0.074 (3)	0.049 (2)	0.033 (2)
C12	0.262 (7)	0.135 (4)	0.056 (2)	0.116 (5)	0.027 (4)	0.020 (3)
C13	0.245 (6)	0.110 (3)	0.060 (2)	0.085 (4)	−0.032 (3)	−0.029 (2)
C14	0.139 (4)	0.103 (3)	0.071 (2)	0.045 (3)	−0.023 (2)	−0.0209 (19)
C15	0.089 (2)	0.084 (2)	0.0754 (18)	0.0332 (16)	−0.0183 (15)	0.0005 (15)
C16	0.119 (3)	0.104 (3)	0.096 (2)	0.046 (2)	0.007 (2)	0.024 (2)

### Geometric parameters (Å, °)

**Table:**

S1—C8	1.662 (2)	C6—C7	1.479 (3)
O1—C7	1.207 (3)	C9—C14	1.370 (5)
N1—C7	1.392 (3)	C9—C10	1.375 (5)
N1—C8	1.394 (3)	C10—C11	1.392 (4)
N1—H1A	0.83 (2)	C10—H10A	0.9300
N2—C8	1.335 (3)	C11—C12	1.341 (7)
N2—C9	1.448 (3)	C11—H11A	0.9300
N2—C15	1.491 (3)	C12—C13	1.354 (8)
C1—C2	1.377 (4)	C12—H12A	0.9300
C1—C6	1.389 (3)	C13—C14	1.418 (7)
C1—H1B	0.9300	C13—H13A	0.9300
C2—C3	1.370 (5)	C14—H14A	0.88 (3)
C2—H2A	0.9300	C15—C16	1.467 (5)
C3—C4	1.364 (5)	C15—H15A	0.9700
C3—H3A	0.9300	C15—H15B	0.9700
C4—C5	1.383 (4)	C16—H16A	0.9600
C4—H4A	0.9300	C16—H16B	0.9600
C5—C6	1.380 (3)	C16—H16C	0.9600
C5—H5A	0.9300		
			
C7—N1—C8	123.84 (19)	C14—C9—C10	119.6 (4)
C7—N1—H1A	116.1 (17)	C14—C9—N2	120.4 (4)
C8—N1—H1A	114.8 (16)	C10—C9—N2	120.0 (3)
C8—N2—C9	122.1 (2)	C9—C10—C11	120.7 (4)
C8—N2—C15	120.0 (2)	C9—C10—H10A	119.7
C9—N2—C15	117.2 (2)	C11—C10—H10A	119.7
C2—C1—C6	119.4 (3)	C12—C11—C10	120.0 (5)
C2—C1—H1B	120.3	C12—C11—H11A	120.0
C6—C1—H1B	120.3	C10—C11—H11A	120.0
C3—C2—C1	120.6 (3)	C11—C12—C13	120.3 (5)
C3—C2—H2A	119.7	C11—C12—H12A	119.8
C1—C2—H2A	119.7	C13—C12—H12A	119.8
C4—C3—C2	120.2 (3)	C12—C13—C14	120.9 (5)
C4—C3—H3A	119.9	C12—C13—H13A	119.5
C2—C3—H3A	119.9	C14—C13—H13A	119.5
C3—C4—C5	120.2 (3)	C9—C14—C13	118.3 (6)
C3—C4—H4A	119.9	C9—C14—H14A	115 (2)
C5—C4—H4A	119.9	C13—C14—H14A	126 (2)
C6—C5—C4	119.9 (3)	C16—C15—N2	110.7 (3)
C6—C5—H5A	120.0	C16—C15—H15A	109.5
C4—C5—H5A	120.0	N2—C15—H15A	109.5
C5—C6—C1	119.7 (2)	C16—C15—H15B	109.5
C5—C6—C7	122.05 (19)	N2—C15—H15B	109.5
C1—C6—C7	118.2 (2)	H15A—C15—H15B	108.1
O1—C7—N1	122.5 (2)	C15—C16—H16A	109.5
O1—C7—C6	122.96 (19)	C15—C16—H16B	109.5
N1—C7—C6	114.58 (18)	H16A—C16—H16B	109.5
N2—C8—N1	115.58 (19)	C15—C16—H16C	109.5
N2—C8—S1	124.27 (16)	H16A—C16—H16C	109.5
N1—C8—S1	120.15 (16)	H16B—C16—H16C	109.5
			
C6—C1—C2—C3	−1.5 (4)	C15—N2—C8—S1	−12.3 (4)
C1—C2—C3—C4	0.8 (4)	C7—N1—C8—N2	−53.4 (3)
C2—C3—C4—C5	0.5 (4)	C7—N1—C8—S1	127.2 (2)
C3—C4—C5—C6	−1.2 (4)	C8—N2—C9—C14	132.1 (3)
C4—C5—C6—C1	0.5 (3)	C15—N2—C9—C14	−57.0 (4)
C4—C5—C6—C7	−176.4 (2)	C8—N2—C9—C10	−51.0 (4)
C2—C1—C6—C5	0.8 (3)	C15—N2—C9—C10	120.0 (3)
C2—C1—C6—C7	177.9 (2)	C14—C9—C10—C11	−1.4 (5)
C8—N1—C7—O1	0.6 (3)	N2—C9—C10—C11	−178.4 (3)
C8—N1—C7—C6	−179.73 (19)	C9—C10—C11—C12	1.9 (5)
C5—C6—C7—O1	143.2 (2)	C10—C11—C12—C13	1.0 (7)
C1—C6—C7—O1	−33.8 (3)	C11—C12—C13—C14	−4.3 (8)
C5—C6—C7—N1	−36.4 (3)	C10—C9—C14—C13	−1.8 (5)
C1—C6—C7—N1	146.6 (2)	N2—C9—C14—C13	175.2 (3)
C9—N2—C8—N1	−21.0 (4)	C12—C13—C14—C9	4.7 (7)
C15—N2—C8—N1	168.3 (2)	C8—N2—C15—C16	102.9 (3)
C9—N2—C8—S1	158.4 (2)	C9—N2—C15—C16	−68.3 (3)

### Hydrogen-bond geometry (Å, °)

**Table:**

*D*—H···*A*	*D*—H	H···*A*	*D*···*A*	*D*—H···*A*
N1—H1A···S1^i^	0.83 (2)	2.62 (2)	3.444 (2)	172 (2)
C4—H4A···O1^ii^	0.93	2.55	3.354 (4)	145

Symmetry codes: (i) −*x*+1, −*y*+2, −*z*+1; (ii) *x*+1, *y*, *z*.
